# Spectroscopic properties of poly(9,9‐dioctylfluorene) thin films possessing varied fractions of β‐phase chain segments: enhanced photoluminescence efficiency via conformation structuring

**DOI:** 10.1002/polb.24106

**Published:** 2016-06-29

**Authors:** Aleksandr Perevedentsev, Nathan Chander, Ji‐Seon Kim, Donal D. C. Bradley

**Affiliations:** ^1^ Department of MaterialsEidgenössische Technische Hochschule (ETH) ZürichVladimir‐Prelog‐Weg 5Zürich8093Switzerland; ^2^ Department of Physics and Centre for Plastic ElectronicsImperial College London, South Kensington CampusLondonSW7 2AZUnited Kingdom; ^3^ Engineering Science and Physics Departments, Mathematical, Physical and Life Sciences DivisionUniversity of Oxford9 Parks RoadOxfordOX1 3PDUnited Kingdom

**Keywords:** conformation, conjugated polymers, microstructure, polyfluorene, spectroscopy

## Abstract

Poly(9,9‐dioctylfluorene) (PFO) is a widely studied blue‐emitting conjugated polymer, the optoelectronic properties of which are strongly affected by the presence of a well‐defined chain‐extended “β‐phase” conformational isomer. In this study, optical and Raman spectroscopy are used to systematically investigate the properties of PFO thin films featuring a varied fraction of β‐phase chain segments. Results show that the photoluminescence quantum efficiency (PLQE) of PFO films is highly sensitive to both the β‐phase fraction and the method by which it was induced. Notably, a PLQE of ∼69% is measured for PFO films possessing a ∼6% β‐phase fraction induced by immersion in solvent/nonsolvent mixtures; this value is substantially higher than the average PLQE of ∼55% recorded for other β‐phase films. Furthermore, a linear relationship is observed between the intensity ratios of selected Raman peaks and the β‐phase fraction determined by commonly used absorption calibrations, suggesting that Raman spectroscopy can be used as an alternative means to quantify the β‐phase fraction. As a specific example, spatial Raman mapping is used to image a mm‐scale β‐phase stripe patterned in a glassy PFO film, with the extracted β‐phase fraction showing excellent agreement with the results of optical spectroscopy. © 2016 The Authors. Journal of Polymer Science Part B: Polymer Physics Published by Wiley Periodicals, Inc. J. Polym. Sci., Part B: Polym. Phys. **2016**, *54*, 1995–2006

## INTRODUCTION

The physical geometry, or *conformation*, of a (semi‐)flexible macromolecule represents an additional parameter space within which its properties and functionalities can be modified. The example of cellulose and starch—two stereoisomers of glucose—is well‐known, while conformational changes are also of fundamental importance to bimolecular interactions of proteins as well as the crystallization behavior of polymers.^1^, ^2^ In the case of conjugated polymers, which typically feature electronic wavefunctions primarily localized onto individual chains,^3^ the chain conformation, as determined by the torsion angles between the constituent molecular subunits, furthermore affects the electronic coupling along the chain and, therewith, a wide range of the resulting optoelectronic properties.^4^, ^5^, ^6^ While lack of control over the formation of conformational isomers and the associated structural polymorphs generally presents an obstacle for achieving consistent solid‐state properties,^7^ there are notable exceptions of conjugated polymers for which well‐defined and reproducible chain conformations are known to exist.

Poly(9,9‐dioctylfluorene) (PFO) is a widely studied conjugated polymer that exhibits a range of attractive properties, such as efficient blue photoluminescence and electroluminescence, high optical gain, and charge‐carrier mobility, as well as good thermal stability, resulting in numerous proposed applications in, among others, light‐emitting diodes (LEDs) and lasers,^8^, ^9^, ^10^, ^11^ sensing,^12^, ^13^ and photonics.^14^, ^15^ In addition to this, the remarkably diverse polymorphism of PFO is also the focus of extensive research effort—both fundamental and application‐driven—with its characteristic well‐defined β‐phase conformational isomer receiving particular attention.^8^, ^9^, ^14^, ^15^, ^16^, ^17^, ^18^, ^19^, ^20^, ^21^, ^22^, ^23^, ^24^, ^25^, ^26^, ^27^, ^28^, ^29^, ^30^, ^31^, ^32^, ^33^

When processed from solutions in good solvents (Hildebrand solubility parameter *δ* ≈ 9.2 cal^1/2^ cm^−3/2^ as for, e.g., chloroform or tetrahydrofuran)^16^, ^17^, ^18^ that also allow for sufficiently rapid solvent evaporation during film deposition (e.g., toluene heated to 100 °C),^15^ in‐plane isotropic, “glassy” PFO films are obtained.^34^ In such films the PFO chains adopt a range of disordered wormlike conformations, collectively also termed “glassy,” featuring a broad distribution of intermonomer torsion angles.^16^, ^23^ By applying the appropriate postdeposition treatments, typically those involving exposure to solvents in liquid^9^, ^15^, ^25^ or vapor^14^, ^18^, ^35^ form, or slowing down the kinetics of film formation via the use of poor or high‐boiling‐point solvents/additives,^21^, ^24^ a fraction of *chain segments* can be driven to adopt the so‐called “β‐phase” conformation. The β‐phase conformation corresponds to a distinct chain‐extended molecular geometry in which the torsion angle between adjacent fluorene units is 180°, resulting in their coplanar orientation, with the octyl substituents located on alternating sides of the backbone.^17^, ^23^, ^29^, ^32^ (See inset of Fig. 2 for the chemical structure of PFO and a schematic illustration of its glassy and β‐phase chain conformations.) By convention, PFO samples containing a fraction of β‐phase chain segments are termed “β‐phase” irrespective of the exact β‐phase fraction.

Due to its extended geometry and the accompanying increase in electronic delocalization, the HOMO‐LUMO transition for the β‐phase conformation corresponds to a substantially *lower* energy (∼0.4 eV) than that characteristic of disordered glassy chains.^18^ Thus, a predominantly glassy PFO sample containing a fraction of β‐phase chain segments represents, in essence, a *self‐doped* material system which offers numerous improvements in optoelectronic performance compared with the baseline glassy samples. For instance, light‐emitting diodes based on a β‐phase PFO active layer displayed a ∼300% increase in the external quantum efficiency, with a concomitant improvement in maximum luminance and color purity for the blue emission.^9^ When induced by blending with a low molecular weight polyphenyl ether, β‐phase PFO exhibits a markedly improved emission stability on annealing in air at elevated temperatures.^26^ In addition, β‐phase PFO dispersed in an inert poly(methyl methacrylate) (PMMA) matrix exhibits enhanced optical gain over a broader spectral region, while allowing for ultrafast (∼300 GHz) optical gain switching.^22^ Furthermore, β‐phase PFO has been proposed as a promising material system for realizing electrically pumped organic lasers.^8^

In the present study, we investigate the spectroscopic properties of PFO thin films possessing varied fractions of chain segments in the β‐phase conformation, with the aim of augmenting the recent advances in understanding and controlling β‐phase formation. First, having shown that solvent‐induced formation of β‐phase occurs via co‐crystallization with the solvent yielding a *polymer‐solvent compound*,^32^, ^33^ we examine the variation of photoluminescence quantum efficiency (PLQE) with both the β‐phase fraction and solvent‐based method by which it was induced. A detailed method of measuring PLQE is presented, with the results expected to allow the performance of β‐phase PFO‐based light‐emitting devices to be further optimized. Second, following the recent demonstration of sub‐micrometer‐scale spatial patterning of the β‐phase, and therewith *refractive index*, in PFO films using dip‐pen nanolithography^15^ we investigate Raman spectroscopy as an alternative means (to optical absorption) for quantifying the β‐phase fraction in PFO films, as well as for spatially resolved imaging of β‐phase patterns. Raman spectroscopy offers the key advantage of being suitable for probing PFO films within device structures for which conventional optical transmission measurements may be problematic due to opaque substrates, the presence of electrodes or strong optical interference effects.

## EXPERIMENTAL

### Materials

PFO, synthesized by Suzuki coupling, was supplied by Cambridge Display Technology and used as received. The polymer had a number‐average molecular weight $M_{n}$ = 18 × 10^3^ g mol^−1^ and a polydispersity index = 2.7, as determined by polystyrene‐equivalent gel permeation chromatography. The absolute molecular weight was calculated by scaling $M_{n}$ down by a factor of 2.7 due to the higher relative chain stiffness of PFO,^17^ giving an estimate of 17 fluorene repeat units per average chain. Toluene (HPLC grade, >99.7%, VWR), decahydronaphthalene (“decalin”; reagent grade, mixture of *cis* and *trans* isomers, Sigma‐Aldrich), cyclohexane (HPLC grade, >99%, VWR), and 2‐(iso‐)propanol (“IPA”; >99.5%, Sigma‐Aldrich) were used as received.

### Thin Film Fabrication

PFO thin films were spin‐coated or drop‐cast onto fused silica substrates (Spectrosil B^®^, UQG Optics Ltd). The processing parameters adopted for fabricating films with varying fractions of β‐phase chain segments are detailed in Table 1. Briefly, to fabricate nominally zero β‐phase (glassy) PFO films, both the PFO solution in toluene and the substrates were placed on a hot‐plate at 100 °C for 2 min immediately prior to spin‐coating. The fabrication of PFO films containing selected fractions of β‐phase chain segments used two related approaches: (i) promoting *slower solvent evaporation* during film deposition (i.e., spin‐coating from unheated solutions or drop‐casting from high boiling‐point solvents) and (ii) postdeposition *swelling* of glassy films with a suitable solvent either by vapor‐annealing or by immersing the films in solvent/nonsolvent mixtures. After deposition and any postdeposition treatments, the films were desiccated over several days to remove any residual solvent. Note that the deposited films were not subjected to further heat‐treatment, thereby avoiding any additional crystallization of the PFO in the so‐called “α‐phase”^36^ and ensuring that the chain ensemble in the films comprised only the glassy and β‐phase conformations.

**Table 1:** Processing Parameters Used for Fabricating PFO Thin Films with Varying Fractions of β‐Phase Chain Segments

β‐Phase Fraction	Solution‐Processing	Postdeposition Treatment
0%	Spin‐coated from 13 mg mL^−1^ solution in toluene at 2300 rpm. Both solution and substrate were placed on a hot‐plate at 100 °C for 2 min immediately prior to spin‐coating.	–
∼0.1%	Spin‐coated from 13 mg mL^−1^ solution in toluene at 2300 rpm, with solution and substrate kept at room temperature throughout.	–
∼1%	As for 0% β‐phase films.	Immersed for 1 min in 1:5 (vol/vol) cyclohexane:IPA mixture.
∼6–8%	As for 0% β‐phase films.	Immersed for 20 s—3 min in 1:2 or 1:3 (vol/vol) cyclohexane:IPA mixtures.
∼10%	As for 0% β‐phase films.	Exposed to saturated toluene vapor at 35 °C for 24 h.
∼26%	Drop‐cast from 0.2 wt % solution in decalin.	Film allowed to slowly dry in a fume hood over several hours.

β‐phase patterns for Raman mapping studies were induced in glassy PFO films by masking parts of the film using a polydimethylsiloxane (PDMS) overlayer followed by immersion in a 1:3 (vol/vol) cyclohexane:IPA mixture for 45 s. The films were subsequently washed in IPA and the PDMS layer was carefully removed to avoid film delamination. A photograph of a typical film is shown in the inset of Figure 7(d). Optical spectroscopy and microscopy confirmed that no dissolution or damage of the films took place.

Film thicknesses were determined using a J. A. Woollam V‐VASE spectroscopic ellipsometer by fitting the Cauchy law to data in the 900–1600 nm nonabsorbing spectral region; all films had a thickness in the 70–80 nm range.

### Optical Absorption and Photoluminescence Spectroscopy

Absorption spectra were recorded with a dual‐beam Shimadzu UV‐2600 spectrophotometer equipped with a diffuse reflectivity (integrating sphere) attachment, allowing the absorption spectra to be corrected for reflection and scattering losses. To correct for specular reflection losses, a clean fused silica substrate was used as a reflection reference; it is negligibly absorbing in the 300–500 nm spectral region, implying that any deviation from 100% transmission can be assumed to be due to reflection. Photoluminescence spectra were recorded in reflection geometry using a Jobin Yvon Horiba Fluoromax‐3 spectrofluorometer (excitation wavelength *λ*_ex_ = 390 nm). Film samples were positioned to provide a ∼75° angle between the excitation beam and the normal to the film plane. All measurements were carried out at room temperature in ambient atmosphere.

### Photoluminescence Quantum Efficiency

PLQE was measured using a Jobin Yvon Horiba Fluoromax‐3 spectrofluorometer equipped with a diffusely reflecting integrating sphere. The use of an integrating sphere ensures that changes in scattering and waveguiding do not markedly affect the light collected by the detector.^37^ An excitation wavelength *λ*_ex_ = 390 nm was used (FWHM bandwidth ≈ 2 nm); all other instrumental settings were kept fixed throughout the measurements. The relative reflectivity of the integrating sphere was measured using a calibrated halogen light source (HL‐2000‐CAL, Ocean Optics). The PLQE value for each sample was calculated from the average of two measurements, with the sample arbitrarily repositioned between successive spectral acquisitions, thereby allowing consecutive measurements to probe different areas of the film and confirm its homogeneity. Further details of the experimental procedure and data analysis are given in the text.

### Raman Spectroscopy and Mapping

Raman spectroscopy was performed using a Renishaw inVia Raman microscope in back‐scattering configuration. The response of the system was calibrated using the 520 cm^−1^ peak of silicon. A HeNe laser provided nonresonant continuous‐wave excitation at 633 nm; unpolarized excitation and detection were used. Excitation light was focused on the top surface of the film using a 50× objective (NA = 0.75). Spectra were routinely acquired from five different areas on each sample in order to confirm their homogeneity on a length scale defined by the area of the excitation laser spot (∼1 μm^2^). The spectra recorded for 10 consecutive acquisitions at the same spot on the sample were averaged; the integration time for each acquisition was 5 s. No major spectral changes occurred for Raman spectra subsequently acquired from the same spot on the film, on the basis of which it was inferred that negligible photo‐degradation arose under these experimental conditions. Were it to do so, it would be evidenced by an overall reduction in the scattered signal, as well as a broadening and shifting to higher wavenumber of the 1606 cm^−1^ mode; such changes are attributable to chain scission and the generation of 9‐fluorenone moieties.^38^

For spectroscopic Raman mapping, the film samples were mounted on a motorized stage and a point‐by‐point mapping method^39^, ^40^ was adopted, in which the samples were raster‐scanned in the *x*/*y*‐axes (5 and 1 μm step sizes, respectively), with Raman spectra recorded at each ( x, y) position. Five consecutive acquisitions with 1 s integration time were averaged to generate each spectrum. All other settings, such as excitation wavelength and optical configuration, were as above. Following averaging, all spectra were background‐corrected by subtracting a linear baseline between 1880 and 980 cm^−^ 1.

## RESULTS

### Optical Absorption and Photoluminescence Spectroscopy

**Figure 1:**
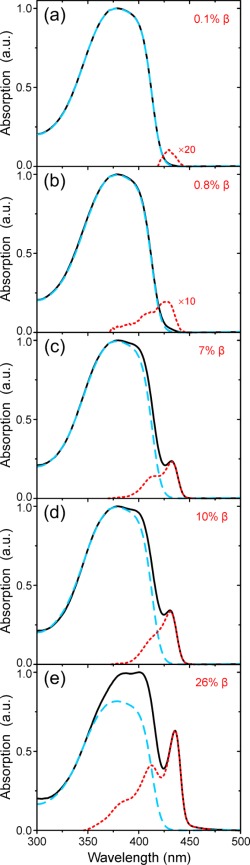
Peak‐normalized absorption spectra for PFO films with increasing [(a) to (e)] fractions of β‐phase chain segments introduced by varied treatments (solid black lines; see Table 1 for details). In each case, the normalized absorption spectrum of a zero β‐phase fraction (i.e., glassy) film of comparable thickness is overlaid (dashed blue lines). The difference spectra correspond to absorption of the β‐phase chain segments (dotted red lines). Taking the ratio of integrated spectral areas allows the relative fraction of β‐phase to be estimated (see text for details); the deduced values are indicated in the top‐right corner of each panel. [Color figure can be viewed in the online issue, which is available at wileyonlinelibrary.com.]

Figure 1 shows the optical absorption spectra of PFO films possessing β‐phase fractions varying from zero (glassy) to 26%. All of the absorption spectra were corrected for specular reflection and scattering losses (see *Experimental*), which can often obscure the finer spectroscopic features, especially for small (<1%) fractions of β‐phase chain segments, and thereby prevent straightforward spectral deconvolution (*vide infra*).

The absorption spectra of β‐phase PFO films are understood to comprise a linear superposition of the contributions from (i) disordered glassy chain segments yielding the broad peak centered at ∼380 nm and (ii) β‐phase segments, the extended geometry of which results in a red‐shifted absorption peak at ∼435 nm.^16^

As a further consequence of its well‐defined (rigid) planar‐zigzag geometry, the subtracted β‐phase absorption spectra exhibit a reduced degree of inhomogeneous broadening compared with the glassy chain ensemble. Hence, the *S*_0_–*S*_1_ vibronic progression of the β‐phase chain segments is well resolved even for β‐phase fractions as low as 1%. Closer inspection shows that the 0‐0 vibronic peak of the *S*_0_–*S*_1_ β‐phase absorption undergoes a minor redshift with increasing β‐phase fraction: from 428 nm (<1% β‐phase) to 432 nm (7% β‐phase) and, finally, 436 nm (26% β‐phase). The nature of this shift remains to be explained; contributing factors are expected to be (i) increasing planarization or, more likely, elongation of the β‐phase chain segments and (ii) changes in the local dielectric environment.

**Figure 2:**
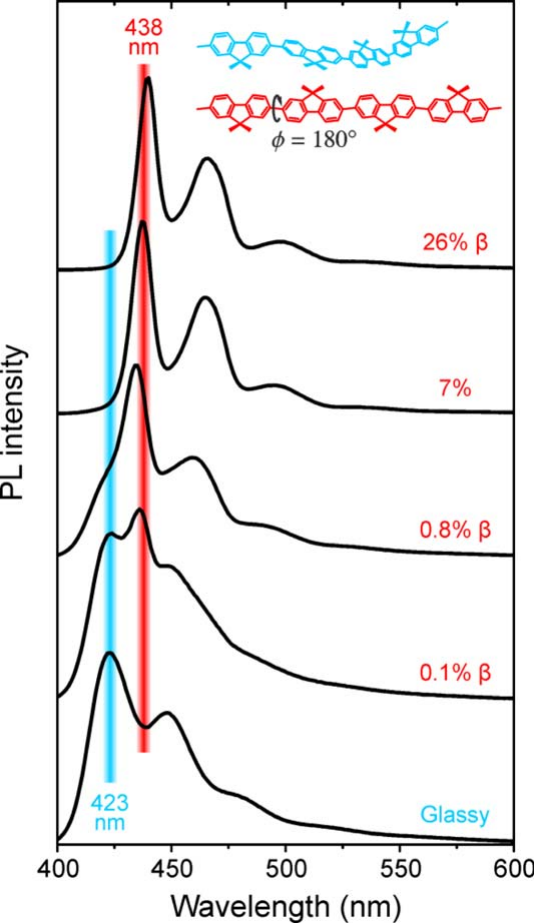
Peak‐normalized PL spectra for PFO films with increasing (bottom to top) β‐phase chain segment fraction. Spectra are offset vertically and the vertical bars (6 nm width) provide guides to the eye for the evolution of the relative intensities of glassy (∼423 nm; blue bar) and β‐phase (∼438 nm; red bar) *S*_1_–*S*_0_ 0‐0 vibronic peaks. The β‐phase segment percentage (deduced from absorption spectra) is indicated for each spectrum. Schematic representations of glassy (blue) and β‐phase (red) chain conformations, with the octyl (C_8_H_17_) side‐chains replaced by methyl groups for clarity, are inset. [Color figure can be viewed in the online issue, which is available at wileyonlinelibrary.com.]

To estimate the fraction of β‐phase chain segments (hereafter simply referred to as “β‐phase fraction”), in each case the absorption spectrum of a zero β‐phase fraction (i.e., glassy) film of comparable thickness was normalized at ∼350 nm, corresponding to the spectral region where absorption by the β‐phase chain segments is expected to be negligible,^16^, ^27^ and subtracted. The difference spectra therefore correspond to absorption of the β‐phase chain segments and are depicted by the dotted red lines in Figure 1. The β‐phase fraction can thus be estimated from the ratio of spectral areas, integrated in the 300–475 nm range, of the difference absorption spectrum, ΔA, and the total absorption of the β‐phase film sample, $A_{total}$, taking into account the relative difference in oscillator strength $f_{osc}$. Previously Huang et al.^28^ used time‐dependent density functional theory to determine that $f_{osc}^{\beta -phase}$ = 1.08 × $f_{osc}^{glassy}$. Consequently, the β‐phase fractions for the PFO films in this study were calculated using eq (1), with the obtained values presented in Figures 1 and 2 and Table 1.

(1) $$\begin{array}{c} \beta -\text{phase}\;\text{fraction}\;(\%)=\\ \frac{\Delta A}{\Delta A+\left\{(A_{\text{total}}-\Delta A)\times 1.08\right\}}\times 100 \end{array}$$

The photoluminescence (PL) spectra for the studied PFO films are shown in Figure 2. The zero β‐phase, glassy film spectrum features *S*_1_–*S*_0_ vibronic peaks at 423, 448, and 476 nm, consistent with previous reports.^20^ Remarkably, the emission from β‐phase chain segments can be resolved even for films with extremely low (∼0.1%) β‐phase fractions which is primarily due to rapid (within a few picoseconds) excitation energy transfer from neighboring higher HOMO–LUMO transition energy, glassy chain segments.^20^, ^21^ This “antenna” effect greatly enhances the sensitivity of the emission to the presence of small fractions of β‐phase chain segments. It is further amplified by spectral diffusion within the ensemble of glassy chain segment site energies, as commonly seen in many conjugated polymer films.^4^

A simple method^31^ was used to deconvolve the glassy and β‐phase contributions in the recorded PL spectra (see Fig. S1 and the accompanying text in the Supporting Information). Table 2 presents a comparison between the β‐phase fraction calculated from the absorption spectra using eq (1) and the deconvolved β‐phase contribution to the PL. The results clearly demonstrate the higher sensitivity of the PL data to β‐phase fraction that, in fact, motivated our subsequent investigation of Raman spectroscopy as an alternative means to quantify the β‐phase fraction in PFO films (*vide infra*). We find that PFO films featuring β‐phase fractions ≥7% yield, in essence, pure β‐phase PL spectra (i.e., without a PL contribution from glassy chain segments) with vibronic peaks located at 438, 465, and 495 nm for the 7% film. Only minor (1–2 nm) spectral variations result thereafter from increasing β‐phase fraction or as a consequence of self‐absorption and/or microstructural heterogeneity.

**Table 2:** Comparison of PFO Film β‐Phase Fractions and Resulting Integrated β‐Phase PL Contributions

β‐Phase Fraction (%)^a^	β‐Phase PL Contribution (%)^b^
0	0
0.1	17
0.8	56
≥7	∼100

a From absorption spectra via eq (1).

b From PL deconvolution.

### Photoluminescence Quantum Efficiency

Photoluminescence quantum efficiency (PLQE) is defined as the ratio of the number of emitted photons to the number of absorbed photons. In an isolated molecule at modest excitation densities this is a function of the excited state electronic structure and the molecular environment. For solid‐state samples, however, PLQE is also strongly dependent, among other things, on microstructure (and correspondingly energetic disorder) due to ensuing variations in excitation energy transfer dynamics and the introduction of alternative radiative/nonradiative decay pathways.^20^ Clearly, maximal values of PLQE are required for enhancing the external efficiency of conjugated‐polymer‐based light‐emitting devices.

Previously Bansal et al.^27^ have reported a decrease of PLQE with increasing β‐phase fraction in PFO films, with the typical PLQE values being 55% (0% β‐phase), 50% (10% β‐phase), and ≤45% (≥30% β‐phase). However, there are a number of factors pertaining to that study that are likely to have affected the accuracy of the reported results. First, it appears that PLQE was determined without explicitly correcting for PL self‐absorption in the integrating‐sphere‐based measurements. Given the large (∼70–100 nm) variation in film thicknesses in that study, the degree of self‐absorption would likely be strongly affected. Second, the β‐phase fraction was estimated from absorption spectroscopy *without* correcting the recorded spectra for scattering and reflectivity losses, thereby producing considerable uncertainty in the reported values.

**Figure 3:**
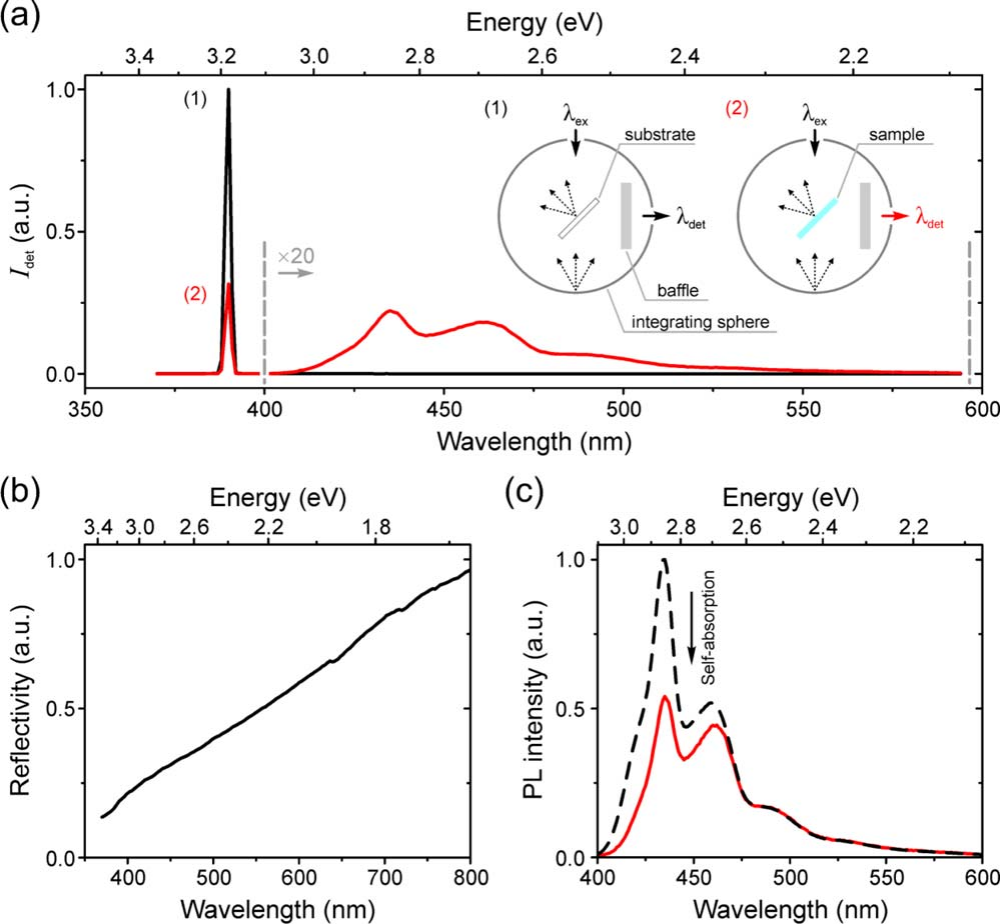
(a) Representative spectral data recorded for a 0.8% β‐phase film [cf. Fig. 1(b)] using the two measurement protocols (see inset and text for details) required for PLQE determination. The detected intensity, $I_{det}$, in the 400–600 nm spectral region is magnified ×20 for clarity. (b) The relative reflectivity spectrum of the integrating sphere measured using a calibrated white‐light source. (c) Comparison of the PL spectra (normalized within their long‐wavelength tails) measured for a 0.8% β‐phase sample in the integrating sphere (solid red line; corrected for sphere reflectivity) and in the standard reflection geometry with the film sample positioned at a high (∼75°) angle of incidence to the excitation light (dashed black line). The increased degree of PL self‐absorption for the integrating‐sphere‐based measurement is evident. The spectral lineshape also shows a residual contribution (weak shoulder on the blue edge of the emission) from glassy phase PFO chain segments. [Color figure can be viewed in the online issue, which is available at wileyonlinelibrary.com.]

With this in mind, PLQE values were determined for the range of β‐phase content PFO films presented above using a modification of the method proposed by Kawamura et al.^41^ This involved two separate measurements [see inset to Fig. 3(a)]. The excitation wavelength was fixed at 390 nm and the detected intensity recorded in the 370–650 nm spectral range. First, a measurement was carried out with the integrating sphere containing a pre‐cleaned fused silica substrate, thereby recording the un‐attenuated intensity of the excitation light [black line and inset (1) in Fig. 3(a)]. Second, a sample was placed in the integrating sphere and the measurement repeated with the same instrumental settings, recording both the reduced intensity of the excitation light and the photoexcited PL intensity [red line and inset (2) in Fig. 3(a)]. The PLQE of the film is then calculated as the ratio of emitted and absorbed photons using eq (2):

(2) $$\text{PLQE}\;(\%)=\frac{\int_{1.91}^{3.14}I_{\text{PL}}^{\left(2\right)}dE}{\int_{3.14}^{3.22}I_{\text{ex}}^{\left(1\right)}dE-\int_{3.14}^{3.22}I_{\text{ex}}^{\left(2\right)}dE}\times 100$$

where $\int I_{ex}dE$ and $\int I_{PL}dE$ are the integrated energy ( E) scale excitation and PL intensities respectively (integration limits are indicated in electron volts), and the superscripts indicate the experimental configuration as in the inset of Figure 3(a). The particular integration limits were chosen in order to selectively integrate the excitation (385–395 nm = 3.22–3.14 eV) and PL (395–650 nm = 3.14–1.91 eV) spectral components.

However, prior to calculating PLQE using eq (2), a number of additional data‐processing steps were undertaken:

- The recorded spectral intensities are first corrected for the wavelength‐dependent reflectivity of the integrating sphere, measured using a calibrated light source [see Fig. 3(b)].
- The reflectivity‐corrected PL intensities are subsequently corrected for self‐absorption using a variation of the method proposed by Ahn et al.^42^ Briefly, this involves normalization by the long‐wavelength tail of the PL spectra recorded for the same sample (i) in the integrating sphere and (ii) in the standard reflection geometry [cf. Fig. 2]; representative data is shown in Figure 3(c). In the latter measurement, PL self‐absorption is minimal due to a high angle of incidence (75°; see *Experimental*) and low film thickness (∼80 nm). The difference between the integrated areas of the two normalized PL spectra then allows for the calculation of a self‐absorption correction factor. The reader is directed to ref. 42 for a full description of the required calculations. We note that this self‐absorption correction method is preferred to the more commonly used procedure first proposed by de Mello et al.^43^ since the latter has subsequently been shown to yield overestimated PLQE values.^42^
- Finally, to correct for the fact that the measurement was made in equal *wavelength* steps while *energy*‐scale integrals were used in eq (2) ( E=hc/λ, where λ is wavelength, h is Planck's constant, and c is the speed of light in vacuum), the reflectivity‐ and self‐absorption‐corrected spectral intensities were multiplied by $\lambda^{2}/hc$ ( dE varies as $hc/\lambda^{2}\;d\lambda$).

**Figure 4:**
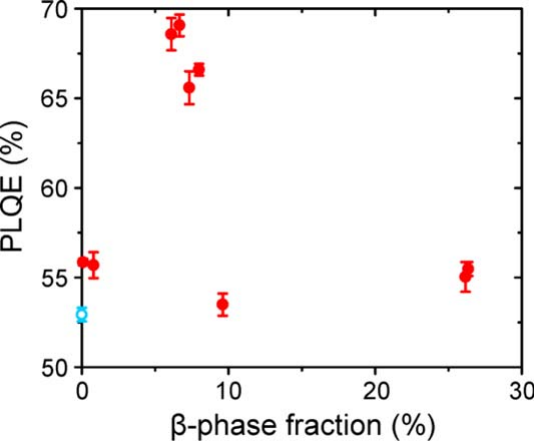
Variation of PFO film PLQE with β‐phase chain segment fraction (filled red circles). The PLQE for a zero β‐phase, “*glassy”* PFO film is also shown (open blue circle). The corresponding error bars are indicated in each case. Films with 0.1% β‐phase were prepared by spin coating from toluene solution; films with 0.8% and 6–8% β‐phase by dipping into solvent/nonsolvent mixtures; films with 10% β‐phase by solvent vapor annealing, and films with ∼26% β‐phase by slow solvent evaporation (see Table 1 for details). [Color figure can be viewed in the online issue, which is available at wileyonlinelibrary.com.]

The resulting variation of PFO film PLQE plotted against β‐phase fraction (albeit using films prepared via a variety of different methods and therefore not necessarily fully comparable) is shown in Figure 4. It can be seen that the PLQE values for most films (i.e., with β‐phase fractions ≤1% and ≥10%) lie in the 53–56% range, only marginally higher than for zero β‐phase (glassy) films, where PLQE ∼53%. Remarkably, however, the PFO films with 6–8% β‐phase fraction, fabricated by dipping into room‐temperature solvent/nonsolvent mixtures (see Table 1), exhibit a substantially higher PLQE. This peaks at ∼69% for the ∼6% β‐phase fraction films and then drops at higher β‐phase fraction. In addition, films prepared via the same method with β‐phase fraction = 0.8% show the more typical PLQE ∼56% confirming that the β‐phase fraction does really matter; the observed variation cannot be simply a microstructural effect arising from different preparation methods.

The reason for this dependence remains unclear and will be further discussed below. We note, however, that the observation of an increased PLQE for β‐phase PFO films fabricated by dipping into solvent/nonsolvent mixtures is consistent with previous reports of enhanced *external* efficiency for LEDs based on similarly structured β‐phase PFO emission layers.^9^

### Raman Spectroscopy

Raman spectroscopy can probe the vibrational “fingerprint” of polymer chains which, in turn, is sensitive to chain conformation due to the associated changes in polarizability and intramolecular force constants. Organic semiconductors typically have large Raman scattering cross‐sections for vibrational modes that are coupled to the π–π* electronic excitations, thus allowing the structural changes within thin (<100 nm) films to be investigated.^44^ Raman spectroscopy has previously been performed on a range of PFO films,^21^, ^45^, ^46^, ^47^, ^48^, ^49^ including those containing a fraction of β‐phase chain segments. However, in general the aforementioned reports only compared the Raman scattering spectra of a *single* β‐phase PFO film with that of a reference zero β‐phase (glassy) film. Therefore, we performed Raman spectroscopy on the range of β‐phase PFO films presented above to systematically investigate the vibrational mode changes as a function of β‐phase fraction.

**Figure 5:**
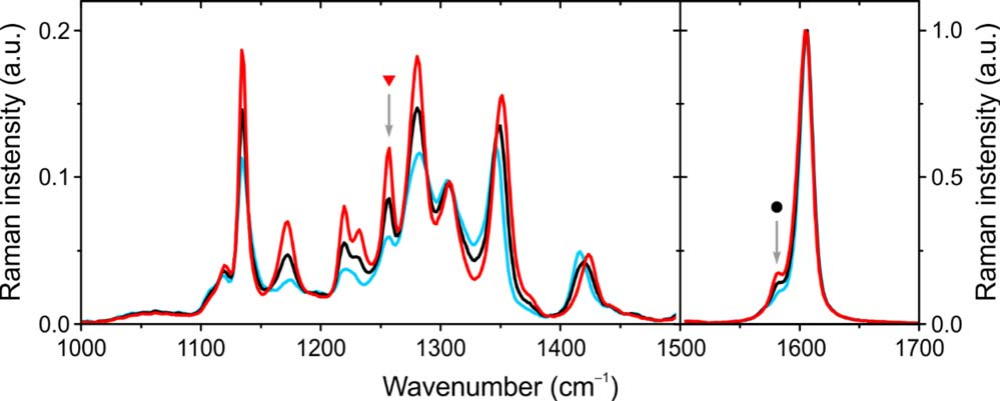
Raman spectra of PFO films with varying β‐phase chain segment fractions: 0% (blue line), 7% (black line), and 26% (red line). The spectra are peak‐normalized by their respective 1606 cm^−1^ modes. Note the different scaling of the two panels adopted for clarity. The arrows indicate the spectral positions of the 1257 cm^−1^ (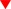) and 1581 cm^−1^ (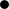) peaks. [Color figure can be viewed in the online issue, which is available at wileyonlinelibrary.com.]

Raman spectra of PFO comprise two principal spectral regions: (i) a low‐wavenumber region (100–700 cm^−^ 1) corresponding predominantly to vibrations of the alkyl side‐chains and (ii) a high‐wavenumber region (1000–1700 cm^−^ 1) dominated by vibrations of the fluorene backbone.^47^, ^48^ Although the low‐wavenumber region can be highly sensitive to backbone conformation due to associated changes in the geometry and vibrational modes of the octyl side‐chains,^47^, ^48^, ^49^ the low signal intensity for this region complicates data interpretation. Hence, our study focused on the high‐wavenumber region, with representative Raman scattering spectra of 0, 7, and 26% β‐phase fraction PFO films shown in Figure 5. The first point to note is that the relative intensity of spectral features in the 1100–1400 cm^−1^ region generally increases with the fraction of β‐phase chain segments. This is partially attributable to increased chain planarity in the β‐phase conformation which, in turn, increases the delocalization of π‐electrons along the conjugated backbone and, hence, the electronic polarizability of the chain segment.^45^ Direct evidence for enhanced polarizability comes from the previously reported DC Kerr effect (electroabsorption spectroscopy) measurements that compared glassy and β‐phase films.^19^

Here, we have chosen to closely examine two specific vibrational modes of PFO, indicated by the arrows in Figure 5. First, the 1257 cm^−1^ mode (indicated by the red triangle), previously assigned to a combination of in‐plane C—H bending and C—C stretching motions of the bond connecting the two phenylene rings within the fluorene moiety.^47^ The intensity of this mode is highly sensitive to the intermonomer torsion angle, increasing with chain planarity.^47^, ^48^ Second, the 1581 cm^−1^ mode (indicated by the black circle) which, although as‐yet not explicitly assigned to any specific vibrational mode, shows a strong increase with β‐phase fraction. This observation is consistent with the preliminary results of our density functional theory (DFT)‐based simulations that provisionally assign it to a backbone‐planarity‐sensitive symmetric C—C ring‐stretching vibration (see Fig. S2 in the Supporting Information). In both cases, we normalize the intensities of the aforementioned modes to the 1606 cm^−1^ symmetric in‐plane C—C ring‐stretching mode, the spectral position of which is found to be virtually insensitive to chain planarity for low β‐phase fractions.^31^, ^45^, ^47^, ^48^, ^49^

**Figure 6:**
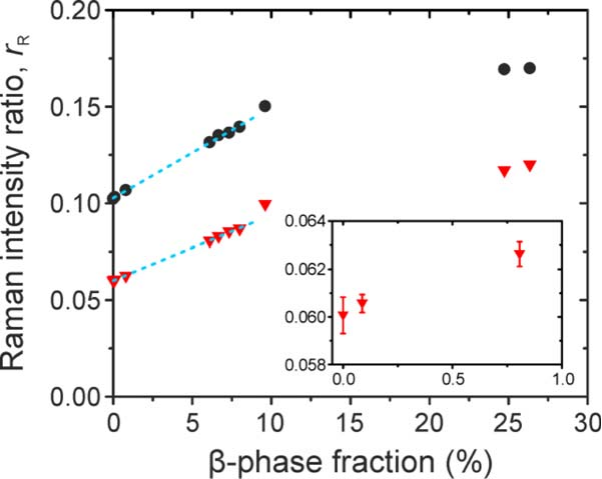
Raman ratios ( $r_{R}$) for the 1257 (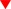) and 1581 (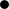) cm^−1^ mode intensities, relative to the 1606 cm^−1^ mode intensity. Dotted lines indicate linear fits to the data for β‐phase fractions ≤9%. Inset shows an expanded view of the 1257 cm^−1^ to 1606 cm^−1^ intensity ratio for β‐phase fractions ≤1%. [Color figure can be viewed in the online issue, which is available at wileyonlinelibrary.com.]

The Raman ratios, $r_{R}$, for the 1257 and 1581 cm^−1^ mode intensities, relative to the 1606 cm^−1^ mode intensity, are shown in Figure 6 as a function of the estimated PFO film β‐phase fraction. For β‐phase fractions ≤9% the variation of each intensity ratio is well described by a linear fit. The inset in Figure 6 further shows that $r_{R}$ is highly sensitive to the presence of β‐phase chain segments and can resolve the presence of very minor, <1%, β‐phase fractions. We speculate that increased chain aggregation for the 10–26% β‐phase fractions,^24^ as well as a possible overestimation of β‐phase fraction for these samples, may be responsible for the deviation from linearity; further studies are needed to test this suggestion. Nevertheless, these results suggest that Raman spectroscopy can provide an alternative means for estimating the fraction of β‐phase chain segments in the 0–9% range when the more common method, namely optical transmission measurements (cf. Fig. 1), cannot (for sample format reasons) be performed.

### Raman Mapping of β‐Phase Patterns

The recently demonstrated spatial patterning of the β‐phase conformation in thin films of PFO^15^ and PFO derivatives^50^ on length scales ≤500 nm has been proposed as an attractive approach to the design and fabrication of novel nanophotonic elements.^14^, ^15^ A versatile imaging method is therefore needed to determine the spatial distribution of β‐phase fraction within such samples to optimize and control the simultaneously induced local change in refractive index.^51^ Direct optical transmission measurements, which have proven to be so effective at extracting the β‐phase fraction for PFO films deposited on transparent fused silica substrates (cf. Fig. 1), are likely to be problematic in the case of complex device architectures involving electrodes and opaque substrates or due to the presence of strong interference effects. PL microscopy, especially when carried out in a confocal geometry with spectrally filtered detection,^15^ may provide a potentially high‐resolution alternative; however, the occurrence of excitation energy transfer prevents straightforward estimation of the β‐phase fraction and its spatial distribution (cf. Table 2 and discussion above). Moreover, concerns may arise over film degradation upon exposure to high‐intensity focused light at resonant UV wavelengths, as generally required for both transmission and PL analysis.

Motivated by the observed linear correlation between the intensity of selected Raman peaks and film β‐phase fraction, we investigated spectroscopic Raman mapping as an alternative versatile means to image β‐phase spatial patterns. This method offers several advantages, namely: (i) lack of any influence from excitation energy transfer, (ii) high sensitivity to β‐phase fraction (<1%; cf. Fig. 6), and (iii) reduced likelihood of film degradation due to nonresonant excitation.

**Figure 7:**
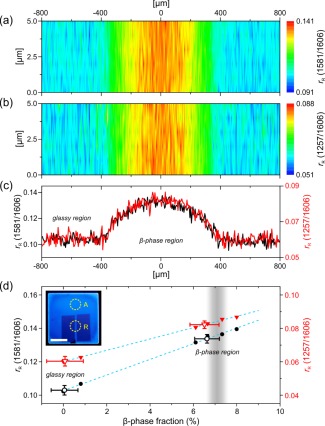
Raman maps of a β‐phase line patterned in a glassy PFO film by masked immersion in a solvent/nonsolvent mixture, recorded as the ratio of Raman intensities, $r_{R}$, at (a) 1581 cm^−1^/1606 cm^−1^ and (b) 1257 cm^−1^/1606 cm^−^ 1. (c) $r_{R}$ profiles across the β‐phase line for the 1581 cm^–1^/1606 cm^–1^ (black line) and 1257 cm^−1^/1606 cm^−1^ (red line) Raman intensity ratios. (d) Experimental data (solid symbols) and linear fits (dotted blue lines) of $r_{R}$ versus β‐phase fraction for the 1581 cm^−1^/1606 cm^−1^ (black circles; left ordinate) and 1257 cm^−1^/1606 cm^−1^ (red triangles; right ordinate) Raman intensity ratios, shown previously in Figure 6. The $r_{R}$ values measured at the center of the β‐phase line and for the surrounding zero β‐phase, “glassy” film (open symbols) are placed on the fit lines, thus allowing the β‐phase fraction to be estimated. The β‐phase fraction (7.1 ± 0.5%) estimated by absorption spectroscopy is indicated by the shaded vertical bar. The inset to (d) shows a photograph of the film under UV illumination (scale bar = 5 mm), with the regions used for Raman mapping and absorption spectroscopy indicated as R and A respectively. The color difference resulting from the PL spectral red‐shift accompanying β‐phase chain segment formation is evident. [Color figure can be viewed in the online issue, which is available at wileyonlinelibrary.com.]

Samples for our proof‐of‐principle study were fabricated by selectively masking as‐spin‐coated, zero β‐phase, glassy films with polydimethylsiloxane (PDMS) overlayers to define an *unmasked* stripe of ∼1 mm width. Subsequent immersion in solvent/nonsolvent mixtures (see *Experimental*) induced β‐phase chain segment formation in the unmasked stripe region. A typical image of the resulting film sample after removal of the PDMS overlayers is shown in the inset of Figure 7(d). Selected‐area PL measurements (not shown here) confirmed that the zero β‐phase (glassy) microstructure was preserved in the masked regions (dark blue rectangles). Further PL spectra were acquired from the unmasked stripe region using the Raman microscope with excitation at *λ*_ex_ = 457 nm and confirmed that it displayed characteristic β‐phase emission (see Fig. S3 in Supporting Information).

Spatial mapping of Raman intensity ratios, $r_{R}$, for the 1257 cm^−1^ and 1581 cm^−1^ modes relative to the 1606 cm^−1^ mode, was performed across the ∼1 mm wide β‐phase stripe in region R of the inset to Figure 7(d). The obtained Raman maps are shown in Figures 7(a,b) with the expected increase in $r_{R}$ evident within the β‐phase stripe. Corresponding $r_{R}$ profiles across the β‐phase stripe are presented in Figure 7(c). Note that the increased noise in the data originates from a reduced integration time per individual “pixel” for the mapping experiments (5 s compared with 50 s for the Raman spectra shown in Fig. 5). The $r_{R}$ profiles for both 1581 cm^−1^/1606 cm^−1^ and 1257 cm^−1^/1606 cm^−1^ ratios are identical and indicate that the β‐phase fraction smoothly increases then decreases across the stripe, following a “semicircular” variation with its maximum value at the stripe‐center. The change in refractive index due to the presence of β‐phase chain segments will follow a similar *graded* profile; an advantageous situation for light propagation since scattering losses are thereby reduced relative to a step index profile.^14^, ^15^

To estimate the maximum β‐phase fraction within the imaged stripe, we revisit the data previously shown in Figure 6 and place the peak $r_{R}$ values obtained for the profiles in Figure 7(c) onto the linear fits of $r_{R}$ as a function of β‐phase fraction. The results shown in Figure 7(d) indicate that comparable maximum β‐phase fractions (6.6 ± 0.4%) are obtained for both Raman intensity ratios. Gratifyingly, this value is in close agreement with the 7.1 ± 0.5% β‐phase fraction deduced from absorption spectroscopy measurements performed on a different unmasked area of the same film [region A in the inset of Fig. 7(d)]. Finally, we note that the average $r_{R}$ values measured *outside* the β‐phase stripe, that is, for the neighboring masked glassy film regions (dark blue rectangles in inset image), correspond to a nominal 0.1 ± 0.5% β‐phase fraction and are therefore fully consistent.

## DISCUSSION

Having observed that the PLQE of PFO films can be strongly influenced by intrachain *structural* modification—specifically showing that the PLQE varied by 15% over a 4% range in β‐phase fraction (cf. Fig. 4)—we now outline a preliminary explanation for this dependence. We speculate that both the *fraction* of β‐phase chain segments and the degree of their *dispersal* in the glassy PFO matrix may be key factors in determining the resulting PLQE.

Since β‐phase chain segments have a propensity to form polymer‐solvent *cocrystals*,^32^, ^33^ the nucleation of β‐phase chain segments and their assembly into domains should depend on a range of processing parameters that affect crystal growth kinetics,^2^ including temperature as well as solvent quality and retained volume.^32^ A variety of β‐phase microstructures is therefore possible (for an illustrative example, the reader is directed to Fig. S4 and the accompanying discussion in Supporting Information).

Previously Virgili et al.^22^ used ultrafast pump‐probe spectroscopy to study thin films of PFO dispersed at 10 wt % into a poly(methyl methacrylate) (PMMA) matrix, specifically focusing on the regions containing well‐dispersed, isolated PFO chains, for which β‐phase‐type PL emission was observed. Compared with reference spin‐coated films of PFO, the isolated PFO chains dispersed in PMMA (and containing a low β‐phase chain segment fraction) were found to exhibit an enhanced stimulated emission that also extended significantly into the long‐wavelength region of the PL spectrum. This behavior was explained as being due to a suppression of charged states that compete with optical gain.

Taken together, these findings strongly suggest that maximal dispersal of β‐phase chain segments favors enhanced luminescence efficiency, provided that the β‐phase fraction is high enough to allow for complete excitation energy transfer from the bulk glassy‐phase chain ensemble. Conversely, aggregated β‐phase clusters are likely to facilitate nonradiative decay, resulting in a reduced PLQE. The latter proposal is supported by a previous report of rapid exciton diffusion within β‐phase domains which will tend to enhance the efficiency of quenching processes.^52^

We emphasise that the above‐proposed explanation is preliminary and additional work will be needed to test more fully the effect of β‐phase fraction and microstructure on emission efficiency. Specifically, the influence of postprocessing method on the PLQE of β‐phase PFO thin films will need to be systematically investigated. An appropriate study might involve a comparison of PLQE values for glassy PFO films exposed at various temperatures to (i) toluene vapor and (ii) liquid toluene:methanol mixtures.^25^ An accompanying polarized PL lifetime analysis^53^ would also be useful to better understand the role of exciton migration.

## CONCLUSIONS

In summary, we have used absorption, photoluminescence and Raman spectroscopy measurements together with PLQE and Raman mode intensity mapping studies to investigate a series of glassy PFO thin films possessing a range of β‐phase chain segment fractions.

Photoluminescence quantum efficiency (PLQE) was shown to be strongly dependent on the fraction of β‐phase chain segments. Notably, PFO films possessing ∼6% β‐phase, induced by immersion in solvent/nonsolvent mixtures, exhibited PLQE ∼69%; a value that is ≥14% higher than the average PLQE of other PFO films with ≤1% and ≥10% β‐phase fractions. A consequence of this observation is that judicious control of the chain conformation should prove a promising approach to increasing the brightness, color purity and external quantum efficiency of PFO‐based light‐emitting devices. Future work will address this opportunity.

Raman spectroscopy measurements revealed a linear correlation between the relative intensity of selected Raman peaks and the β‐phase fraction determined by absorption measurements; this linearity held for samples with 0–9% β‐phase chain segment fractions, flattening off at higher values. This suggests that Raman spectroscopy could be suitable to estimate β‐phase fractions within device structures, for which conventional analysis based on optical transmission measurements is problematic. The generality of this linear relationship remains, however, to be established, for instance in respect of varying PFO molecular weight and β‐phase chain segment induction method. A proof‐of‐principle demonstration of using spectroscopic Raman mapping to image mm‐scale β‐phase patterns in glassy PFO films was also presented, with the extracted β‐phase fractions showing excellent agreement with the results of optical spectroscopy. Confocal Raman mapping is expected, therefore, to become an important tool in the optimization of submicrometer‐scale β‐phase patterns for a range of emerging photonic applications.^15^

Finally, it is intriguing to note the extent to which conformational control remains an under‐utilized means to optimize conjugated polymer function for a variety of applications. It is our expectation that such control will increasingly play an important role, albeit that the necessary levers to achieve conformational control remain to be more generally established.

## Supporting information

Supporting Information
