# Supplementary material for: Spectroscopic properties of poly(9,9‐dioctylfluorene) thin films possessing varied fractions of β‐phase chain segments: enhanced photoluminescence efficiency via conformation structuring
Source: J Polym Sci B Polym Phys. 2016 Jun 29;54(19):1995–2006. doi: 10.1002/polb.24106 (PMC5347961; doi:10.1002/polb.24106)
Supplement: Supplementary file 1 — Supporting Information [file POLB-54-1995-s001.pdf]

Supporting Information

“Spectroscopic Properties of Poly(9,9-dioctylfluorene) Thin Films Possessing Varied Fractions of β-phase Chain Segments: Enhanced Photoluminescence Efficiency via Conformation Structuring”

Aleksandr Perevedentsev e*t al.*

1. Deconvolution of PL spectra of β-phase PFO thin films

We performed PL deconvolution[1] to determine the contribution of β-phase chain segments to the PL emission of the studied PFO thin films and to correlate these results with the β-phase fractions extracted using optical absorption spectroscopy (cf. Figure 1 in the main text). The PL deconvolution procedure we adopted is illustrated in Figure S1 and involves fitting the PL spectra measured for β-phase PFO films (cf. Figure 2 in the main text) with a sum of two reference PL spectra:

(i) PL of *β-phase* PFO (dashed red lines in Figure S1), obtained by photoexcitation at 432 nm, corresponding to the spectral position of peak absorption by the β-phase chain segments. This reference spectrum was recorded for a PFO film featuring a ~7% β-phase fraction to ensure sufficiently well-resolved spectral features.

(ii) PL of *glassy* PFO (dashed blue lines in Figure S1), measured for a zero β-phase, glassy PFO film with photoexcitation at 390 nm.

The relative magnitudes of the two constituent reference spectra were then adjusted in order for their sum (solid green lines in Figure S1) to fit the overall PL spectrum measured for a given β-phase PFO film. Keeping in mind that self-absorption can lead to strong PL attenuation at the low-wavelength side of the spectrum, the fitting was done only in the 440–650 nm spectral region.

The relative PL contribution of β-phase chain segments (shown in Table 2 in the main text) was then estimated from the ratio of integrated intensities of the corresponding fitted β-phase and sum PL spectra. The integration was also carried out only in the 440–650 nm spectral region. Given that the Huang-Rhys factor is higher for the glassy films, this procedure will tend to underestimate the β-phase contribution.

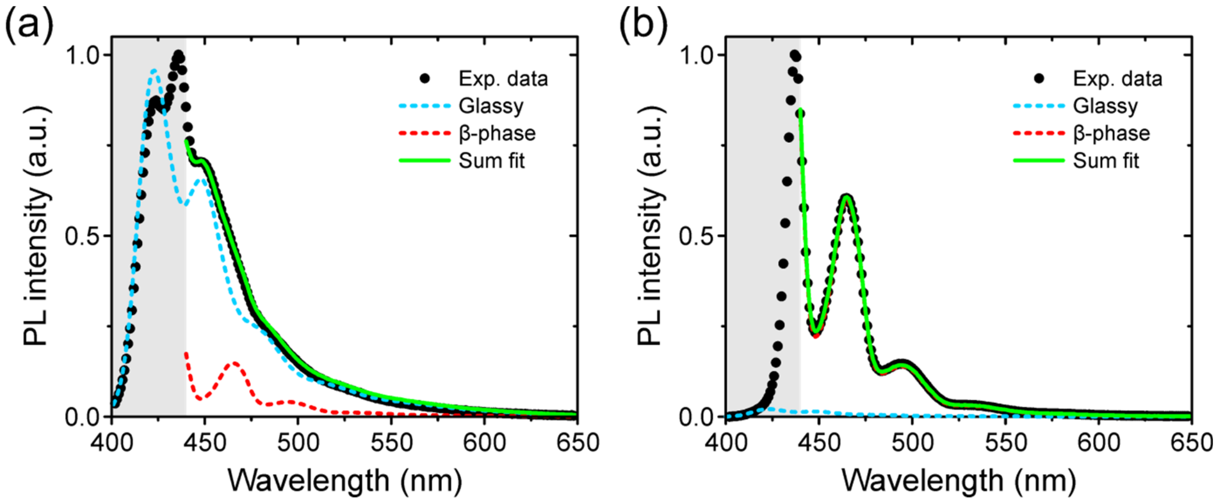

Figure S1. Deconvolution of PL spectra of PFO thin films possessing **(a)** 0.1% and **(b)** 7% β-phase fractions as determine by optical absorption spectroscopy. PL spectra recorded with photoexcitation at 390 nm are shown by symbols (●) and the reference glassy and β-phase PFO PL spectra are shown by blue and red dashed lines, respectively. The sum fits (green lines) show good agreement with the experimental data in the 440–650 nm spectral region (at wavelengths beyond the shaded area in the graph).

2. Simulation of Raman spectra using density functional theory (DFT) calculations

Density functional theory (DFT) allows the modelling of chemical structures to give an insight into their properties. For quantum chemical calculations, the polyfluorene structures were optimised and their Raman spectra calculated using a B3LYP (Becke's three-parameter hybrid) functional and the 6-31G(d,p) basis set as used in previous reports.[2,3] This combination was found to provide a good balance between computational time and accuracy.[4–8] A scaling factor of 0.988 was used for the calculated frequencies, in close agreement with the value used by Irikura *et al.*, who reported that a scaling factor of 0.9627 ± 0.0403 gives optimum results.[9] The GAUSSIAN09 software package was used for all quantum chemical calculations.

In order to streamline the calculations, simulations were performed for a PFO pentamer (five fluorene repeat units), corresponding to a short segment of the chains in the actual polymer sample used in this study (average length = 17 repeat units; see *Experimental* section in the main text). Furthermore, since the vibrational modes in the 1600 cm^{-1} region correspond to the PFO backbone, the simulated fluorene pentamer was disubstituted with methyl (instead of octyl) side-chains. DFT was used to optimise the chain geometry, following which a frequency analysis was performed to identify the Raman-active vibrational normal modes; these were found to be in close agreement with the experimental data.

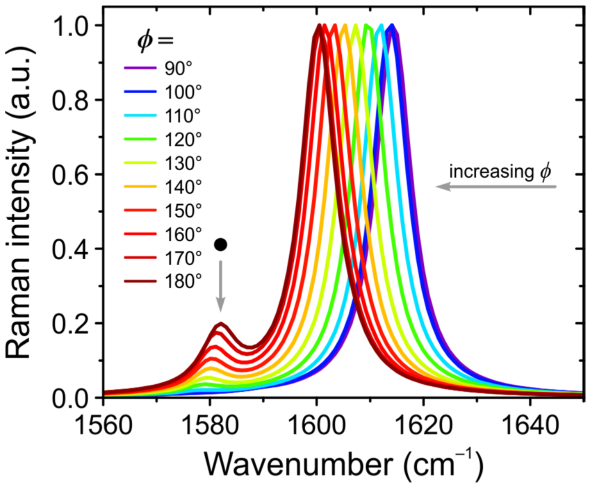

Figure S2. Raman spectra of fluorene pentamers (di-substituted with methyl side-chains) simulated via DFT calculations for a range of inter-monomer torsion angles ϕ. The position of the peak at 1581 cm^{-1} for ϕ = 180° is indicated by the arrow.

Figure S2 shows that increasing planarity of the backbone (that is, increasing ϕ) leads to a softening of the dominant symmetric C-C ring-stretching mode by ~14 cm^{-1} relative to that for orthogonal monomers ($\phi = 90°$). Such behaviour has been attributed to increasing effective conjugation along the backbone, accompanied by a reduction in intra-molecular force constants.[2,3] For glassy PFO film samples with β-phase fractions <30% the dominant symmetric C-C ring-stretching mode is essentially static at 1606 cm^{-1} (see Fig. 5 in the manuscript text)[2,10,11] suggesting that for longer conjugation lengths the sensitivity of this mode *frequency* to conjugation is rather limited and hence of little use in our study.

However, it is also seen in Figure S2 that as the inter-monomer torsion angle is increased from 90° to 180° the mode at ~1581 cm^{-1} increases in relative intensity (and hardens by ~3 cm^{-1}). In addition, these intensity changes persist for glassy PFO film samples with β-phase fractions increasing up to 30% (see Fig. 5 in the manuscript text). The relative ~1581 cm^{-1} mode intensity thus provides an alternate (backbone-planarity/polarizability-sensitive) symmetric C-C ring-stretching mode-based measurement with which to monitor β-phase chain segment growth.[11] Fig. 6 in the manuscript text suggests that saturation of the intensity change does eventually onset but only for β-phase fractions above ~10%.

Finally, we note that our analysis (cf. Fig. 6 in the main text) uses the relative intensity at a *fixed* (1581 cm^{-1}) spectral position. This may at first glance appear at odds with the observed hardening of this mode for increasing ϕ (see Fig. S2). However, the β-phase conformation is understood to be inherently well-defined, corresponding to a chain segment for which ϕ = 180°, rather than a distribution of inter-monomer torsion angles.

Other Raman modes also show relative intensity changes with β-phase fraction (see Figure 5 in the manuscript text), sometimes accompanied by complicating frequency shifts, linewidth changes and so on. The 1257 cm^{-1} mode, previously assigned to a combination of in-plane C-H bending and C-C stretching motions of the bond connecting the two phenylene rings within the fluorene moiety[2,3,10] shows a relatively clear trend and so was also used to monitor β-phase fraction, showing good linearity up to ~10% (cf. Fig. 6 in the main text).

3. Raman mapping and localised PL excitation with the confocal Raman spectrometer

Prior to mapping studies of the solvent/non-solvent mixture immersed PFO films, test Raman spectra were recorded for the PDMS-masked and non-masked areas. No evidence was found that the masking process had any effect on the PFO film other than the desired frustration of β-phase chain segment formation.

In addition, when an excitation wavelength λ_{ex} = 457 nm was selected instead of the λ_{ex} = 633 nm used for all other measurements it was found to be possible to excite β-phase PL. This suggests that the excitation at λ_{ex} = 457 nm provides sufficient overlap with the β-phase long-wavelength absorption (peak at ~433–435 nm, FWHM ≈ 14 nm, depending on the exact β-phase fraction).

The PL vibronic peak wavelengths, λ_{PL}, were determined from the obtained spectra as:

$$\lambda_{\mathrm{PL}} = \left(\frac{1}{\lambda_{\mathrm{ex}}} - \bar{\nu}\right)^{-1}$$

where λ_{ex} is the excitation wavelength (457 nm) and $\bar{\nu}$ is the measured peak wavenumber.

As shown in Figure S3, the spectrum recorded for the region of the film exposed to the solvent/non-solvent mixture (see Figure 7(d) in the main text) features two peaks at 467 and 500 nm which correspond, respectively, to the S_1-S_0 0-1 and 0-2 vibronic peaks of the β-phase PL emission spectrum. Conversely, excitation of the masked area of the film does *not* produce an evident PL contribution, again fully consistent with the PDMS overlayer being an effective way of preventing β-phase formation during immersion in solvent/non-solvent mixtures.

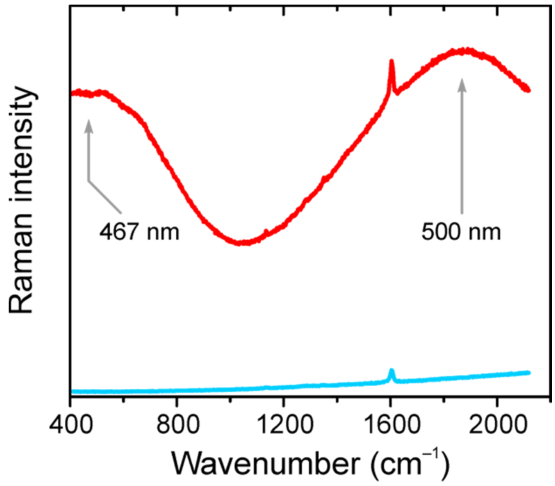

Figure S3. Spectra recorded with excitation wavelength λ_{ex} = 457 nm for the masked (blue line) and unmasked (red line) regions of the PFO film following exposure to a solvent/non-solvent mixture. The wavelengths corresponding to the observed peaks are indicated. Also evident in both spectra is the PFO symmetric C-C ring-stretching Raman mode at 1606 cm^{-1}.

4. Microstructure and PLQE of β-phase PFO films

Having shown that solvent-induced formation of β-phase chain segments occurs via co-crystallisation with the solvent, yielding a *polymer-solvent compound*,[12,13] it is expected that the well-understood factors that determine the kinetics of nucleation and crystal growth will govern its progress. Figure S4 shows the classical bell-shaped crystal growth rate curve which originates from a superposition of (i) a nucleation term that *increases* with the degree of undercooling and (ii) a mobility term which *reduces* with the degree of undercooling.[14] For illustrative purposes, the dipping and solvent vapour annealing methods are placed at the two extreme positions on the crystal growth rate curve based on their respective processing temperatures in order to highlight the expected differences in microstructure of comparable (8 and 10%) β-phase fraction films prepared by these two methods.

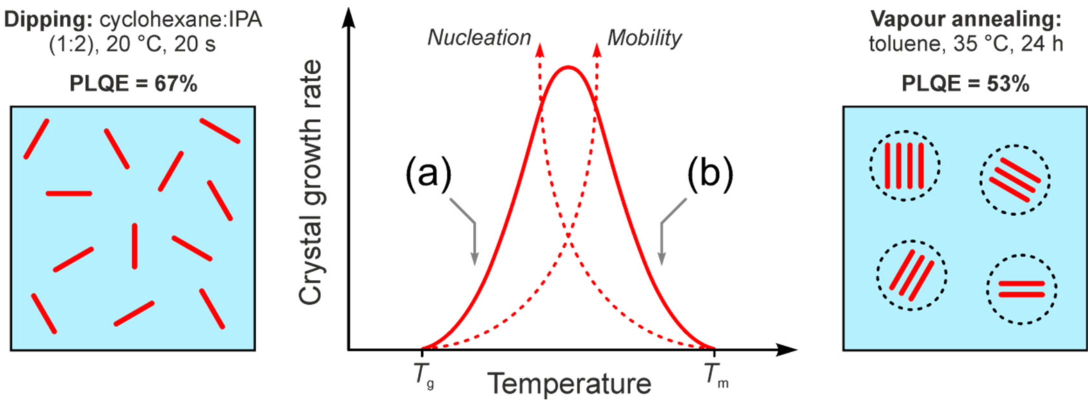

Figure S4. Schematic illustration of crystal growth rate as a function of temperature (solid red line) originating from a superposition of the nucleation and mobility terms (dashed red lines, as indicated). The expected positions of the solvent-based **(a)** dipping and **(b)** vapour annealing methods to generate comparable fractions of β-phase chain segments are indicated. The corresponding schematic illustrations show the relative dispersals of β-phase chain segments (red lines; equal number in both samples) in otherwise glassy PFO films (blue squares) that may be expected for the two methods.

In the case of the dipping method performed at a *lower* (20 °C) temperature, the *nucleation* term dominates; hence, the resulting microstructure can be expected to feature *well-dispersed* β-phase chain segments. The correspondingly reduced chain mobility at lower temperatures would, however, hinder the tendency for chain aggregation. We further note that the lower overall solvent quality in the case of the dipping method ($\delta \approx 10.4$ cal$^{1/2}$ cm$^{-3/2}$ for (1:2) cyclohexane:IPA mixture as compared with $\delta \approx 8.9$ cal$^{1/2}$ cm$^{-3/2}$ for toluene used for vapour annealing) would also favour a high nucleation density for β-phase chain segments.

Conversely, in the case of vapour annealing with toluene performed at a *higher* (35 °C) temperature the *mobility* term is expected to dominate. Since the resulting β-phase fraction for the two methods was comparable (8–10%), the microstructure of the vapour-annealed films is expected to feature *aggregated* β-phase clusters, the formation of which is facilitated by both increased chain mobility and the significantly longer (24 h; see *Experimental*) processing time.

Upon photoexcitation of the bulk glassy chains (as was done for the PLQE measurements; see *Experimental*) the β-phase segments/domains would be rapidly populated by efficient excitation energy transfer.[15] However, it is plausible that the larger size of β-phase clusters in vapour-annealed PFO films would additionally allow for subsequent migration-activated or charged-state-induced non-radiative quenching within the β-phase clusters which may then be responsible for the reduced PLQE values.

References

[1] A. Perevedentsev, S. Aksel, K. Feldman, P. Smith, P. N. Stavrinou, D. D. C. Bradley, *J. Polym. Sci., Part B: Polym. Phys*. **2015**, *53*, 22.

[2] M. Arif, C. Volz, S. Guha, *Phys. Rev. Lett.* **2006**, *96*, 025503.

[3] C. Volz, M. Arif, S. Guha, *J. Chem. Phys*. **2007**, *126*, 064905.

[4] A. D. Becke, *J. Chem. Phys*. **1993**, *98*, 5648.

[5] F. J. Devlin, J. W. Finley, P. J. Stephens, M. J. Frisch, *J. Phys. Chem*. **1995**, *99*, 16883.

[6] A. D. McLean, G. S. Chandler, *J. Chem. Phys*. **1980**, *72*, 5639.

[7] R. Krishnan, J. S. Binkley, R. Seeger, J. A. Pople, *J. Chem. Phys*. **1980**, *72*, 650.

[8] L. A. Curtiss, M. P. Mcgrath, J. P. Blaudeau, N. E. Davis, R. C. Binning, L. Radom, *J. Chem. Phys*. **1995**, *103*, 6104.

[9] K. K. Irikura, R. D. Johnson, R. N. Kacker, *J. Phys. Chem. A* **2005**, *109*, 8430.

[10] M. Ariu, D. G. Lidzey, M. Lavrentiev, D. D. C. Bradley, M. Jandke, P. Strohriegl, *Synth. Met*. **2001**, *116*, 217.

[11] N. Chander, Doctoral Thesis: Imperial College London, United Kingdom, **2016**.

[12] A. Perevedentsev, P. N. Stavrinou, D. D. C. Bradley, P. Smith, *J. Polym. Sci., Part B: Polym. Phys.* **2015**, *53*, 1481.

[13] A. Perevedentsev, P. N. Stavrinou, P. Smith, D. D. C. Bradley, *J. Polym. Sci., Part B: Polym. Phys.* **2015**, *53*, 1492.

[14] L. H. Sperling, *Introduction to Physical Polymer Science*; Wiley: New Jersey, **2006**.

[15] A. L. T. Khan, P. Sreearunothai, L. M. Herz, M. J. Banach, A. Köhler, *Phys. Rev. B* **2004**, *69*, 085201.
